# Medical Eras

**Published:** 1847-02

**Authors:** 


4. *Medical Eras.*—Every fresh event is dignified by the name of ERA, now-a-days; we seem to have arrived at the *era of eras.* There are the MESMERIAN era, the HAHNEMANNIAN era, the PRIESSNITZIAN era, and at length we have, as we said on a former occasion, the quintessence of all in the FORBESIAN era—not to mention the DICKSONIAN, the MORRISONIAN, the ELLIOTSONIAN, the RASPAILIAN, and others of a kindred spirit. In the midst of the age of eras, why should not *we* lay claim to one? We must do so in self-defence. Ours, then, shall be the ANTI-QUACKERY ERA.

We have begun a task, and will go on with it. We are already in the second year of *our* era. We have devoted one year to this subject, and are willing to devote a life to it, if so be this hydra may be beheaded and seared. But the profession must assist us. They must no longer enact the wagoner calling upon Jupiter; they must put their own shoulders to the wheel.

We have dwelt on the injuries accruing to public morals, public health, and the interests of the profession, from the vices of quackery. We have dwelt, too, upon the numerous modes in which professional men may and do, sometimes inconsiderately, sometimes from questionable motives, second the influence of this social Moloch. We have pointed out some of the evils arising from testimonials in favor of quack nostrums, from the system of prescribing quack medicines, and from the by no means singular evil of medical men appearing as the proprietors of such medicines, and from the system of allowing the most nefarious quacks to take what liberties they please with, and to convert to any vile uses, the names of men known and eminent in the medical profession.

The wrongs wrought at the Royal Society we have exposed, and will expose again. But the profession ought to feel the question as their own, and raise their voice as one man against such enormities. Now that we are attacking *the quackeries*, exposing the knavery of the duping and the folly of the duped, we emphatically say, let the profession help us, or rather let them help themselves, for the cause is their own. Medicine is indeed *militant* in these days, and every true son of medicine should be a soldier in defence of his profession.

But, aided or unaided, we will drive all the *isms*, and all the *eras*, the GENUS of quacks, and the essense and quintessence of quackery, from our good and beneficent profession, into their own regions of darkness and crime, with their own nicknames of "drug doctors" and "drug diseases," for their fiendish consolation; and we beseech every member of our profession to recoil from touching either their works or the works in which they are given shelter, as they would shun the viper, its venom, and its nest; and to shun their very words, terms, and descriptions, as profane and obscene words are shunned by the excellent of the earth.

Wherever labor, effort, talent, originality, striving to raise the status, by raising the science, of our profession, be discovered, let the members of that profession be prompt to express its applause,—let calumny, whether in our conversations, or in our critical labors, cease,—let the dignity of our profession be upheld,—let all wrong, and let all quackery, in or out of it, be eschewed.

If there be a name in the profession which we ought to revere, it is that of Louis; for labour, for probity, for talent, that physician is without a parallel. Did our readers ever peruse the first few pages of the "Examen de l'Examen?" We will one day lay these before them. It is a perfect picture of a great and good man—of a high-minded and conscientious physician. His writings, like his noble physiognomy, have the sternness of inflexible integrity and truth; his heart is filled with the love of mankind, and devotion to his profession. But what shall we say of the Louis critics, who are striving to turn his authority against medicine,—striving to prove from him, for instance, that bleeding is of no value in pneumonia, and, therefore, neither in rheumatism, nor in acute inflammation of any kind. Some defender of Louis, who shall rescue him from his slanderers, is required. Everywhere we are met with perversions of the facts and results of Louis, in favour of quackery, of "nature," of the "vis medicatrix," and the other excuses of quackish empiricism.

To return to our *era*. Let us establish a code of ethics,—let us be tender of the reputation of our profession, and of our professional brother—let calumny cease,—let our conduct be ethical, our opinions logical; the former implies every honorable feeling; the latter, laborious research, accuracy, probity, and a profound hatred of all quackery, whatever its parentage or its name, whether within or without the pale of the profession. Let us do justly, foster the right and the truth, repudiate wrong, iniquity, and falsehood, with all slander against our profession, all calumnies, and all nicknames. Let the profession, and let every individual thereof, be true to itself and himself.—*Lancet*, in *Med. News*.

